# Prognostic Value of Vimentin Is Associated With Immunosuppression in Metastatic Renal Cell Carcinoma

**DOI:** 10.3389/fonc.2020.01181

**Published:** 2020-08-04

**Authors:** Jia xi Yao, Xiang Chen, Yan jun Zhu, Hang Wang, Xiao yi Hu, Jian ming Guo

**Affiliations:** Department of Urology, Zhongshan Hospital, Fudan University, Shanghai, China

**Keywords:** metastatic renal cell carcinoma, prognosis, immunohistochemistry, vimentin, immunosuppression

## Abstract

**Introduction:** Vimentin, a classical marker of epithelial–mesenchymal transition, reflects the invasiveness of cancer cells. Its role in the genesis and progression of tumor has been reported in various cancers, including renal cell carcinoma. However, the impact of vimentin on tumor microenvironment, particularly its implication with tumor-infiltrating immune cells, is unknown.

**Methods:** We conducted this study in 231 patients with metastatic renal cell carcinoma (mRCC) to determine the potential relationship between vimentin and immune status. Using immunohistochemical staining, expression of vimentin, CD8, FOXP3, programmed cell death protein 1 (PD-1), and programmed cell death ligand 1 (PD-L1) were evaluated in resected tumor tissue. Kaplan–Meier analysis and Cox regression models were used for survival analysis. Chi-square test, Fisher exact test, and Mann–Whitney *U*-test were used for comparison between vimentin high and low groups.

**Results:** High expression of vimentin, stroma PD-L1, and PD-1 indicated poor overall survival, whereas low regulatory T cell or high CD8+ T cell infiltration indicated long overall survival. Stroma PD-L1 (*P* = 0.030), vimentin (*P* = 0.026) expression, and CD8^+^ T cell infiltration (*P* < 0.001) were independent prognostic factors in mRCC. High vimentin expression was accompanied by high PD-1, PD-L1 expression, and increased regulatory T cell infiltration (all *P* < 0.001), indicating immunosuppression in the tumor microenvironment.

**Conclusions:** We revealed that vimentin expression was associated with immunosuppression in mRCC, and the immune-suppressive status might be possibly posed by PD-1/PD-L1. Patients with high vimentin expression may acquire potential benefit from the recently approved PD-1/PD-L1 inhibitors. However, further clinical trials are needed to validate our findings.

## Introduction

Renal cell carcinoma (RCC) is one of the most common urological cancers, which accounts for 2–3% of all malignant tumors in adults (1). Radical surgery is effective in localized RCC, but once metastasized, the prognosis is poor. The median survival of patients with recurrence and metastasis is only about 26 months treated with sunitinib (2). Currently, tyrosine kinase inhibitor (TKI) is one of the first-line agents for metastatic RCC (mRCC), with a response rate of <30% (3). With the development of a variety of targeted drugs and sequential TKI therapy regimen, survival of mRCC patients is prolonging. Most of these patients have received multi-line TKI therapy and lack new available drugs (4). Studies have shown abundant immune cell infiltration in the RCC microenvironment. Thus, interferon-alpha and interleukin-2 were used to treat RCC (5). Immunotherapy with immune checkpoint inhibitors (monoclonal antibodies) of programmed cell death protein 1 (PD-1)/programmed cell death ligand 1 (PD-L1) has also shown a good effect in recent trials, particularly in high-risk mRCC (6).

Epithelial–mesenchymal transition (EMT) contributes to the proliferation, invasion, and metastasis of carcinoma (7). EMT makes epithelial cells lose their polarity, decrease cell-to-cell and cell-to-extracellular matrix adhesion, and increase the invasiveness of tumor cells (8). Vimentin, a component of mesenchymal cell skeleton, increased expression during the EMT process. Previous studies have suggested that vimentin indicates EMT, which affects tumor progression by increasing the invasive ability of the tumor (9). Also, it is closely related to the ability of tumor invasion and metastasis. Currently, studies in malignant tumors have shown that vimentin plays an important role in cell cycle regulation, migration, adhesion, and EMT of carcinoma (10). The latest report shows that vimentin or EMT can affect the infiltration of immune cells in tumor microenvironment (11).

Whether EMT is associated with anti-tumor immunity in RCC is yet unknown. In this study, expression of vimentin, immune checkpoint molecules (PD-1 and PD-L1), tumor-infiltrating CD8^+^ T cells, and regulatory T cells (Tregs) were assessed by immunohistochemical staining to reflect immune status in RCC. Also, we aimed to evaluate the prognostic value of vimentin expression and its association with the tumor immune environment in an mRCC cohort.

## Materials and Methods

### Clinical Data and Specimen Collection

Patients with mRCCs from Zhongshan Hospital, Fudan University were enrolled in the study from June 2007 to June 2017. All patients received first-line sunitinib or sorafenib therapy, and characteristics are summarized in Table 1. Clinical data and follow-up state were collected. Progression-free survival (PFS) was evaluated from the introduction of system therapy to the date of progression, and overall survival (OS) was from the introduction of system therapy to the date of death or last follow-up. The follow-up period ranged from 3 to 111 months (median 26 months). No patients received preoperative radiotherapy or chemotherapy. The ethics committee of Zhongshan Hospital, Fudan University, approved the study, and all patients signed informed consent.

**Table 1:** Clinical and pathologic characteristics of patients according to vimentin expression.

**Characteristics**	**Patients**		**Vimentin expression in mRCC**		
	***n***	**%**	**Low**	**High**	***P*-value**
No of patients	231	100	107	124	
Sex					0.158
Male	169	73.2	75	94	
Female	62	26.8	34	28	
Age					0.119
≤ 57 years	110	47.6	46	64	
>57 years	121	52.4	63	58	
Histologic type					**0.020**
Clear cell type	199	86.1	100	99	
Non-clear cell type	32	13.9	9	23	
Fuhrman grade					**0.022**
2	112	48.5	62	51	
3 + 4	119	51.5	47	71	
Initial TNM stage					0.142
Stage I + II	126	54.5	65	61	
Stage III + IV	105	45.5	44	61	
Pulmonary metastasis					**0.021**
Present	143	61.9	76	67	
Absent	88	38.1	33	55	
Metastatic organ number					0.615
1	153	66.2	74	79	
≥2	78	33.8	35	43	
Tumor hemorrhage/necrosis					**0.002**
Present	74	32	24	50	
Absent	157	68	85	72	
IMDC					0.197
Favorable	38	16.5	23	15	
Intermediate	153	66.2	68	85	
Poor	40	17.3	18	22	
Tumor size					0.331
≤ 4 cm	41	17.7	24	17	
>4 and ≤ 7 cm	95	41.1	44	51	
>7 and ≤ 10 cm	60	26	24	36	
>10 cm	35	15.2	17	18	
Systemic therapy					0.825
Sunitinib	136	58.9	65	71	
Sorafenib	95	41.1	44	51	
Response					0.957
PR	51	22.1	25	26	
SD	118	51.1	55	63	
PD	62	26.8	29	33	

*TIMC, tumor-infiltrating mononuclear cells; TC, tumor cells; IMDC, International Metastatic Renal Cancer Database Consortium; TNM, tumor–nodes–metastases; mRCC, metastatic renal cell carcinoma; PR, partial response; SD, stable disease; PD, progressive disease. Bold value indicates statistical significance*.

### Tissue Samples and Immunohistochemical Staining

Archival anonymized, formalin-fixed, paraffin-embedded (FFPE) tumor tissue samples obtained from nephrectomy or tumor biopsy specimens of patients were enrolled in this study. Immunohistochemical staining was performed as previously described (12). A total of five immunohistochemistry (IHC) assays were developed with serial sections: Primary antibodies used were mouse anti-human CD8 (ab17147, Abcam, concentration 1:400), mouse anti-human FOXP3 (ab20034, Abcam, concentration 1:100), rabbit anti-human vimentin (ab92547, Abcam, concentration 1:200), mouse anti-human PD-1 (ab52587, Abcam, concentration 1:100), and rabbit anti-human PD-L1 (ab205921, Abcam, concentration 1:400). In total, 231 individual patient formalin-fixed, paraffin-embedded blocks were selected for IHC staining of PD-L1, PD-1, vimentin, CD8, and FOXP3. IHC-stained slides were reviewed and evaluated by two independent investigators; the mean of two evaluations was recorded as the final score for each specimen. Staining intensity was classified as negative for 0 points, weakly positive for 1 point, positive for 2 points, and strongly positive for 3 points. The PD-L1 expression on both stromal and tumor cells were calculated. Staining intensity score multiplied the percentage of positive cells (1 point for 1 percentage) to get the final PD-L1 H-score. Vimentin expression was assessed using a similar H-score semiquantitative method as PD-L1. Final immunohistochemical scores were staining intensity multiplied to positive cell percentage. The percentage of vimentin-positive cells was scored 1 point for positive cell percentage ≤ 10%, 2 points for percentage 11 to 40%, 3 points for percentage 41 to 75%, and 4 points for percentage ≥75%. Scores of 0 to 7 were considered low expression and scores of 8 to 12 considered high expression for vimentin (cut-off by median) (13). Five independent areas of each slide were examined under a microscope to calculate the counts of FOXP3^+^, CD8^+^, or PD-1^+^ tumor-infiltrating cells. Median of Treg density (16/mm^2^), CD8^+^ cell density (143/mm^2^), and PD-1^+^ cell density (82/mm^2^) were set as the cutoff point for high and low infiltration.

### Statistical Analysis

Chi-squared test, Fisher exact test, and Mann–Whitney *U*-test were performed to compare qualitative and quantitative parameters. Kaplan–Meier curves were compared using the log-rank test. Univariate and multivariable Cox proportional hazards models were used to explore the potential predictive effect of the biomarker (vimentin, CD8^+^ T cell density [high/low], or PD-L1, PD-1 status [+/–]), and other characteristics. Variables with *P* < 0.1 in the univariate analysis entered the multivariate model. A two-sided *p* < 0.05 was considered statistically significant. All statistical analyses were performed using SPSS 21.0 software (SPSS Inc., Chicago, IL, USA).

## Results

### Association of Vimentin and Clinical Characteristics of Metastatic Renal Cell Carcinoma Patients

**Figure 1:**
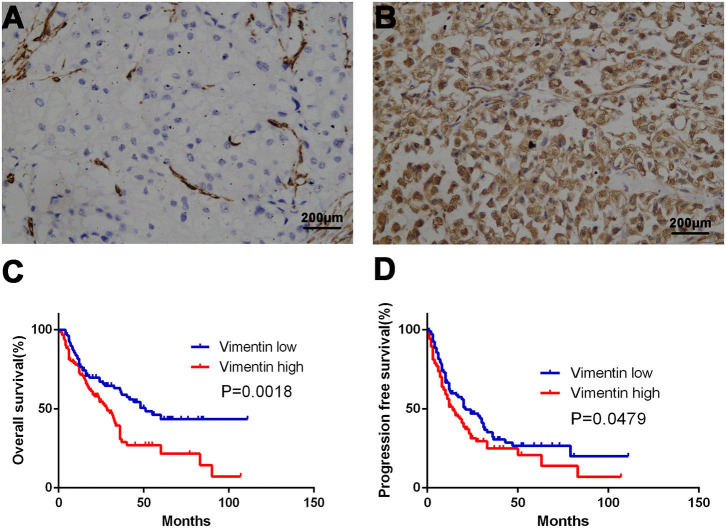
Association of vimentin expression and prognosis. Typical images of low vimentin expression **(A)** (negative expression in tumor cells and positive expression in endothelial cells) and high expression in tissue microarray. **(B)** Kaplan–Meier curve of overall survival (OS) **(C)** and progression-free survival (PFS) **(D)** according to vimentin expression. Original magnification: ×100. *P*-value calculated by log-rank test.

A total of 231 cases were enrolled, 169 males and 62 females, aged from 14 to 87 years old; mean age was 57.2 years old. Typical vimentin immunostaining is shown in Figures 1A,B. Overall, 46.32% (*n* = 107) of mRCC cases were considered to have low vimentin expression, whereas 53.68% (*n* = 124) of mRCC cases were considered to have high vimentin expression. Characteristics of low or high vimentin expression group are summarized and compared in Table 1. The expression of vimentin in 231 cases of mRCC had no significant relation with sex, age, and initial tumor–nodes–metastases stage. With the Fuhrman grade increasing, the expression of vimentin also increased. High vimentin expression was associated with high Fuhrman grade (*P* = 0.022), non-clear-cell histologic type (*P* = 0.020), absent of pulmonary metastasis (*P* = 0.021), and tumor hemorrhage/necrosis (*P* = 0.002).

### Association of Vimentin and Immune Status in Metastatic Renal Cell Carcinoma

**Figure 2:**
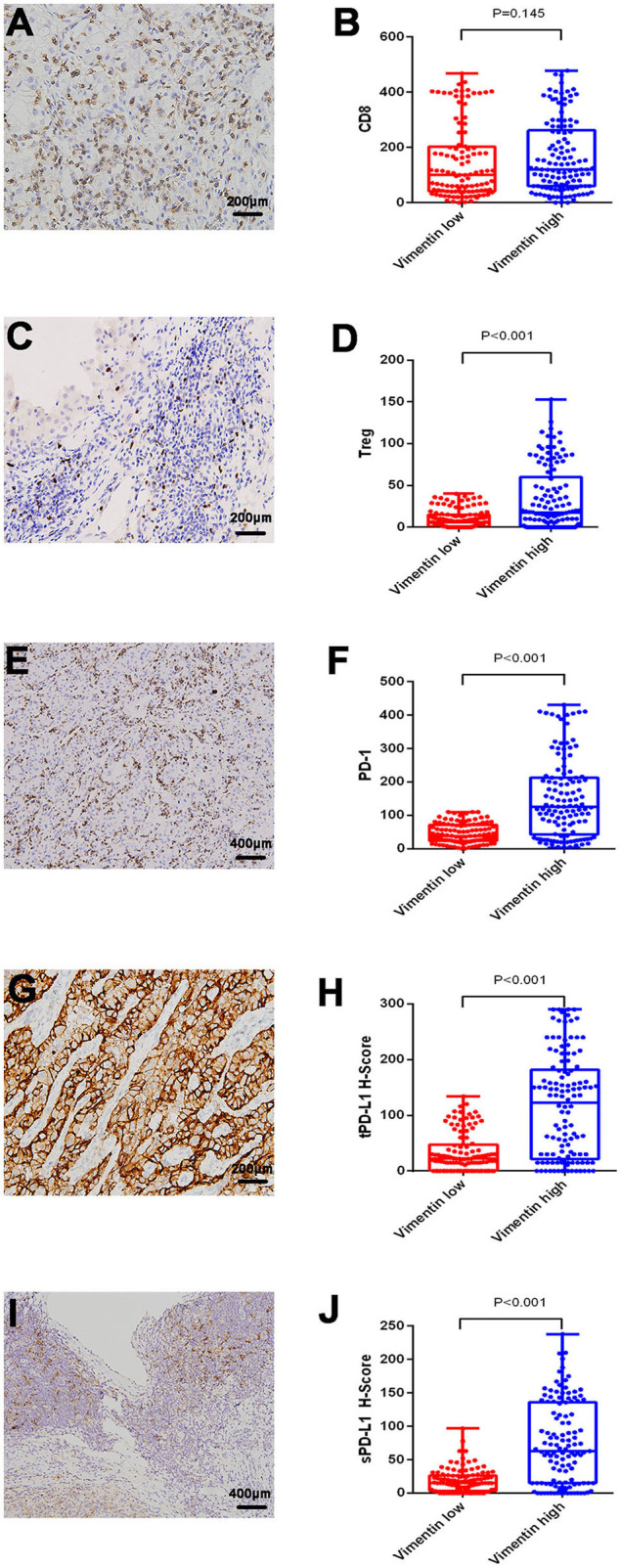
Association of vimentin expression and immune status. Typical images of infiltrating CD8^+^ T cell **(A)** and regulatory T cell (Treg) **(C)**. Typical images of programmed cell death protein 1 (PD-1) expression **(E)**, tumor PD-L1 (tPD-L1) **(G)**, stroma PD-L1 (sPD-L1) **(I)**. CD8^+^ T cell **(B)**, and Treg **(D)** infiltrating levels in vimentin low and high groups. PD-1 **(F)**, tPD-L1 **(H)**, and sPD-L1 **(J)** expression levels in vimentin low and high groups.

Typical images of tumor PD-L1 (tPD-L1), stroma PD-L1 (sPD-L1), and PD-1 staining are shown in Figures 2E,G,I. PD-1 and PD-L1 (both sPD-L1 and tPD-L1) overexpression were associated with high vimentin expression (Figures 2F,H,J; all *P* < 0.001). Typical images of tumor-infiltrating CD8^+^ T cells and Tregs are also shown in Figures 2A,C. The association between vimentin expression and CD8^+^ T cell infiltration number was statistically insignificant (Figure 2B; *P* = 0.145). However, high vimentin expression was significantly associated with increased Treg infiltration (Figure 2D; *P* < 0.001).

### Impact of Vimentin and Immune Status on Prognosis of Metastatic Renal Cell Carcinoma

**Figure 3:**
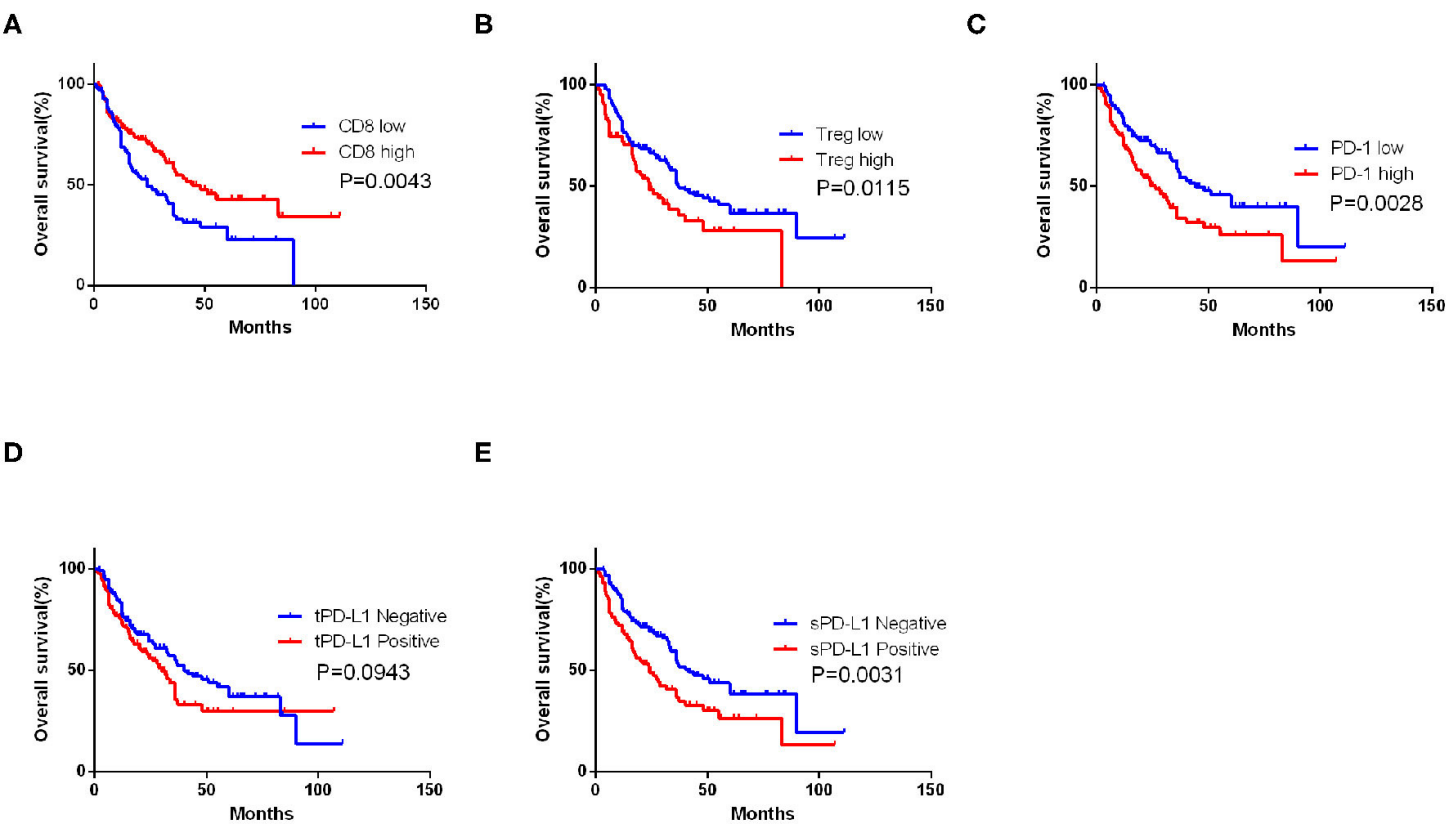
Impact of immune features on overall survival (OS) of metastatic renal cell carcinoma (mRCC) patients. Kaplan–Meier curve of OS according to the CD8^+^ T cell **(A)**, Treg infiltrating number **(B)**, programmed cell death protein 1 (PD-1) **(C)**, tumor PD-L1 (tPD-L1) **(D)**, and stroma PD-L1 (sPD-L1) **(E)** expression in mRCC patients. *P*-value calculated by log-rank test.

Univariate analysis demonstrated that vimentin, sPD-L1, PD-1 expression, and Treg, CD8^+^ T cell infiltration were possible prognostic factors for OS. Clinical factors of initial tumor–nodes–metastases stage, pulmonary metastasis, and International Metastatic Renal Cancer Database Consortium risk group were possible prognostic factors for OS as well. Multivariate analysis indicated that vimentin expression [hazard ratio (HR) = 1.697, 95% confidence interval (CI): 1.067–2.699, *P* = 0.026] was an independent prognostic factor for OS of the patients of mRCC, along with sPD-L1 expression (HR = 1.602, 95% CI: 1.046–2.452, *P* = 0.030) and CD8^+^ T cell infiltration (HR = 0.456, 95% CI: 0.305–0.679, *P* < 0.001, Table 2). Clinical factors of pulmonary metastasis and International Metastatic Renal Cancer Database Consortium risk group remained to be independent prognostic factors of OS (*P* = 0.003 and *P* < 0.001, respectively). Kaplan–Meier survival analysis also showed that patients with high vimentin, sPD-L1, and PD-1 expression had significantly poorer OS (*P* = 0.0018, *P* = 0.0031, and *P* = 0.0028, respectively, Figures 1C, 3C,E). Patients with low Treg infiltration or high CD8^+^ T cell infiltration showed longer OS than those with high Treg infiltration or low CD8^+^ T cell infiltration (*P* = 0.0115, *P* = 0.0043, respectively, Figures 3A,B). No association were observed between OS and tPD-L1 expression (*P* = 0.0943; Figure 3D). Although high vimentin expression indicated poor PFS in Kaplan–Meier survival analysis (cut-off by median, *P* = 0.0479, Figure 1D), it was not an independent prognostic factor for PFS (Table S1).

**Table 2:** Univariate and multivariate Cox regression analyses for OS of patients (*n* = 231).

**Variables**	**Univariate**		**Multivariate**	
	**HR (95% CI)**	***p*-value**	**HR (95% CI)**	***p*-value**
Age				
>57 years vs. ≤ 57 years	1.226 (0.850–1.767)	0.276		
Sex				
Male vs. female	0.787 (0.511–1.211)	0.275		
Histologic type				
Non-clear cell vs. clear cell type	1.370 (0.846–2.218)	0.201		
Fuhrman grade		**0.037**		0.093
2	1.000		1.000	
3	1.332 (0.891–1.992)		0.939 (0.606–1.457)	
4	1.932 (1.158–3.221)		1.715 (0.956–3.077)	
Initial TNM stage		**0.023**		0.059
I	1.000		1.000	
II	1.009 (0.566–1.800)		1.200 (0.653–2.205)	
III	1.265 (0.833–1.920)		1.259 (0.797–1.990)	
IV	2.668 (1.399–5.085)		2.573 (1.305–5.073)	
Tumor size				
Per 1 cm increase	1.273 (0.884–1.832)	0.195		
Pulmonary metastasis				
Present vs. Absent	1.702 (1.146–2.527)	**0.008**	1.945 (1.263–2.995)	**0.003**
Systemic therapy				
Sorafenib vs. Sunitinib	1.059 (0.719–1.560)	0.772		
Tumor necrosis				
Present vs. Absent	1.306 (0.887–1.924)	0.176		
Metastatic organ number				
≥2 vs. 1	1.065 (0.726–1.563)	0.746		
tPD-L1				
Positive vs. negative	1.363 (0.943–1.968)	0.099	0.736 (0.452–1.198)	0.217
sPD-L1				
Positive vs. negative	1.712 (1.189–2.465)	**0.004**	1.602 (1.046–2.452)	**0.030**
Treg				
High vs. low	1.611 (1.105–2.351)	**0.013**	1.371 (0.899–2.090)	0.143
CD8				
High vs. low	0.593 (0.411–0.855)	**0.005**	0.456 (0.305–0.679)	**<0.001**
PD-1				
High vs. low	1.733 (1.199–2.504)	**0.003**	1.474 (0.956–2.272)	0.079
Vimentin				
High vs. low	1.800 (1.234–2.628)	**0.002**	1.697 (1.067–2.699)	**0.026**
IMDC		**<0.001**		**<0.001**
Favorable	1.000		1.000	
Intermediate	1.474 (0.816–2.663)		1.572 (0.849–2.911)	
Poor	3.710 (1.935–7.113)		3.869 (1.930–7.757)	

*tPD-L1, tumor cells PD-L1 expressions; sPD-L1, stromal immune cells PD-L1 expressions; CI, confidence interval; IMDC, International Metastatic Renal Cancer Database Consortium; OS, overall survival; TNM, tumor–nodes–metastases; HR, hazard ratio. Bold value indicates statistical significance*.

## Discussion

Vimentin is a canonical marker of EMT. It is an important regulator of cellular motility, ubiquitously expressed in normal mesenchymal cells to maintain cellular integrity (14). Increased vimentin expression has been reported in various cancers, such as prostate cancer, gastric cancer, and esophageal squamous cell carcinoma, and correlates with tumor growth, invasion, migration, and poor prognosis (15–17). Williams et al. (18) also found that expression of vimentin was closely related to low differentiation, strong invasiveness, and easy metastasis in chromophobe RCC. We confirmed that high expression of vimentin indicated poor OS in this mRCC cohort mainly composed of patients with clear-cell RCC.

CD8^+^ T cell is the main effector of anti-tumor immunity. PD-1/PD-L1 acts as inhibitory molecules reflecting the immunosuppressive state in tumor microenvironment (19). PD-1- or PD-L1-positive Tregs inhibit the expansion of immune response and reduce anti-tumor immunity in microenvironment (20). Exhaustion of CD8^+^ T cell, characterized by increased expression of inhibitory checkpoint receptors, is a gradual loss of the effector function and the proliferation potential (21). Overexpression of PD-L1 on tumor cells can inhibit cytotoxicity of CD8^+^ T cells (22). PD-1/PD-L1 pathway also affects the suppressive properties of the Tregs (23). The blockade of the PD-1/PD-L1 pathway prevents the conversion of naive Th cells to a Treg phenotype and rejuvenates CD8^+^ T cells (24). Therefore, PD-1/PD-L1-targeted immune checkpoint inhibitors have been approved for the treatment of advanced melanoma, kidney cancer, head and neck cancer, and non-small cell lung cancer (25). Consistent with the previously mentioned theories, CD8^+^ T cell was associated with favorable survival, whereas PD-1, sPD-L1, and Tregs were associated with unfavorable survival in our cohort.

Whether vimentin is related to immune status in the microenvironment of RCC has not been reported yet. Asgarova et al. (26) reported that EMT can induce PD-L1 promoter demethylation and upregulate the expression of PD-L1, which may be one of the possible mechanisms of vimentin-induced immunosuppression. It has also been reported that vimentin is involved in the migration of immune cells, such as lymphocytes (27). Our study indicated that high expression of vimentin in mRCC was associated with immunosuppressive status. Patients with high vimentin expression were accompanied by high expression of sPD-L1, tPD-L1, and PD-1. These patients also had more plentiful Treg infiltration (*P* < 0.001), but CD8^+^ T cell infiltration was comparative (*P* = 0.145). Because Tregs and PD-1/PD-L1 play immunosuppressive roles in the microenvironment, high levels of these factors indicate an immunosuppressive status. Although the number of infiltrating CD8^+^ T cells was similar in patients with vimentin high and low expression, the function of CD8^+^ T cells was probably inhibited.

Tumor-derived PD-L1 is capable of immunosuppression, but we suppose that stromal-derived PD-L1 plays a more important role in CD8^+^ T cell inhibition because sPD-L1 has direct contact with CD8^+^ T cells. In accordance with our speculation, sPD-L1 was an independent prognostic factor for OS, whereas tPD-L1 was not. sPD-L1 mainly expresses on the surface of Tregs and myeloid-derived suppressor cells (28). We did not evaluate infiltration of myeloid-derived suppressor cells in this cohort because the marker of this cell population for immunohistochemical staining is not available at present. For Tregs (marked by FOXP3), high infiltration suggested poor survival in our cohort. As the receptor of PD-L1, high PD-1 expression also indicated poor survival. Results of univariate COX regression analysis were concordant with survival analysis. After adjusting for various clinical and immunological factors in multivariate COX regression model, sPD-L1, vimentin, and CD8^+^ T cells remained to be independent prognostic factors.

A previous study has found a deleterious impact of vimentin expression on clinical outcome of localized RCC (29). Our study extended this conclusion to an mRCC cohort. Also, our results further suggested the association between vimentin expression and immune-suppressive status. Recruitment of Tregs, but not CD8^+^ T cells, may be affected by tumor vimentin expression. High vimentin expression may also induce increased PD-L1 expression both in tumor and stromal cells. However, the mechanisms behind the phenomenon were still vague. In addition to its well-known role in EMT, vimentin was also suggested to have another possible tumor-promoting mechanism in our study: it may induce immunosuppression via the PD-1/PD-L1 axis and inhibit CD8^+^ T cell function. With the fast development and application of PD-1/PD-L1-targeted immunotherapy in oncology, patients with high vimentin expression mRCC may be appropriate candidates due to accompanied high PD-1/PD-L1 expression.

There are several limitations to this study. First, this retrospective study was conducted in a single center with limited sample size; external validation with a prospective design is needed to confirm our findings. Second, all mRCC patients included in our study received sunitinib/sorafenib as first-line treatment. The results of our study should be generalized with caution for patients receiving other therapy, particularly for those receiving immune checkpoint inhibitors. Third, relationships between other EMT markers, such as E-cadherin, N-cadherin, fibronectin, etc., and immunosuppression status were not investigated in this study, and the association of vimentin and PD-1/PD-L1 axis was only demonstrated in human tissue level. The underlying mechanism should further be confirmed in an intervention experiment. Fourth, intra-tumor heterogeneity may bias our evaluation of vimentin expression. For example, the center of the tumor and the invasive border may have a different level of vimentin expression.

In summary, the present study confirmed in mRCC that high vimentin expression indicated poor OS. Vimentin expression positively associated with PD-1, PD-L1 expression, and Treg infiltration. In addition to EMT, vimentin may induce immunosuppression via PD-1/PD-L1. Patients with high vimentin expression may have a potential benefit from the recently approved PD-1/PD-L1 inhibitors.

## Data Availability Statement

The raw data supporting the conclusions of this article will be made available by the authors, without undue reservation, to any qualified researcher.

## Ethics Statement

The study was performed according to the principles of the Declaration of Helsinki. The study was approved by the ethics committee of Zhongshan Hospital, Fudan University and all patients signed informed consent.

## Author Contributions

Acquisition of data, analysis, interpretation of data, statistical analysis, and drafting of the manuscript were carried out by JY, XC, and YZ. YZ and HW provided technical and material support. XH and JG were responsible for the study concept and design, analysis and interpretation of data, drafting of the manuscript, obtained funding, and study supervision. All authors read and approved the final manuscript.

## Conflict of Interest

The authors declare that the research was conducted in the absence of any commercial or financial relationships that could be construed as a potential conflict of interest.
