# Supplementary material for: Prognostic Value of Vimentin Is Associated With Immunosuppression in Metastatic Renal Cell Carcinoma
Source: Front Oncol. 2020 Aug 4;10:1181. doi: 10.3389/fonc.2020.01181 (PMC7417332; doi:10.3389/fonc.2020.01181)
Supplement: Supplementary file 1 [file Table_1.DOC]

| **Table S1. Univariate and multivariate Cox regression analyses for PFS of patients (n=231)** | | | | |
| --- | --- | --- | --- | --- |
| **Variables** | **Univariate** | | **Multivariate** | |
|  | **HR (95% CI)** | ***p*-value** | **HR (95% CI)** | ***p*-value** |
| Age |  |  |  |  |
| >57 years *vs* ≤57 years | 1.101 (0.790-1.534) | 0.570 |  |  |
| Gender |  |  |  |  |
| Male *vs* Female | 0.737 (0.500-1.086) | 0.122 |  |  |
| Histologic type |  |  |  |  |
| Non-clear cell *vs*  clear cell type | 1.212 (0.762-1.929) | 0.418 |  |  |
| Tumor nucleus grade |  | 0.310 |  |  |
| 2 | 1.000 |  |  |  |
| 3 | 1.011 (0.703-1.454) |  |  |  |
| 4 | 1.445 (0.885-2.361) |  |  |  |
| Initial TNM stage |  | **0.011** |  | **0.033** |
| I | 1.000 |  | 1.000 |  |
| II | 1.033 (0.617-1.729) |  | 1.072 (0.635-1.810) |  |
| III | 1.096 (0.750-1.601) |  | 1.091 (0.737-1.613) |  |
| IV | 2.746 (1.491-5.057) |  | 2.549 (1.354-4.796) |  |
| Tumor size |  |  |  |  |
| Per 1cm increase | 1.087 (0.917-1.289) | 0.334 |  |  |
| Pulmonary metastasis |  |  |  |  |
| Present vs Absent | 1.587 (1.114-2.261) | **0.010** | 1.581 (1.086-2.302) | **0.017** |
| Systemic therapy |  |  |  |  |
| Sorafenib vs Sunitinib | 0.823 (0.582-1.164) | 0.270 |  |  |
| Tumor necrosis |  |  |  |  |
| Present vs Absent | 1.224 (0.857-1.748) | 0.267 |  |  |
| Metastatic organ number |  |  |  |  |
| ≥2 *vs* 1 | 1.018 (0.717-1.447) | 0.919 |  |  |
| tPD-L1 |  |  |  |  |
| Positive *vs* Negative | 1.263 (0.904-1.766) | 0.172 |  |  |
| sPD-L1 |  |  |  |  |
| Positive *vs* Negative | 1.499 (1.075-2.090) | **0.017** | 1.295 (0.885-1.895) | 0.184 |
| Treg |  |  |  |  |
| High *vs* Low | 1.451 (1.026-2.050) | **0.035** | 1.373 (0.957-1.969) | 0.085 |
| CD8 |  |  |  |  |
| High *vs* Low | 0.723 (0.519-1.007) | 0.055 | 0.597 (0.417-0.854) | **0.005** |
| PD-1 |  |  |  |  |
| High *vs* Low | 1.543 (1.106-2.154) | **0.011** | 1.448 (0.961-2.181) | 0.077 |
| Vimentin |  |  |  |  |
| High *vs* Low | 1.392 (0.995-1.946) | 0.053 | 1.155 (0.767-1.738) | 0.491 |
| IMDC |  | **0.024** |  | **0.019** |
| Favorable | 1.000 |  | 1.000 |  |
| Intermediate | 1.060 (0.665-1.693) |  | 1.042 (0.639-1.697) |  |
| Poor | 1.861 (1.066-3.250) |  | 1.938 (1.076-3.489) |  |
| Abbreviations: tPD-L1,tumor cells PD-L1 expressions; sPD-L1, stromal immune cells PD-L1 expressions; CI, confidence interval; IMDC, International Metastatic Renal Cancer Database Consortium; PFS, progression free survival. | | | | |
